# Cost-effectiveness analysis of pembrolizumab vs. chemotherapy as second-line treatment for advanced esophageal carcinoma in the United States

**DOI:** 10.3389/fpubh.2022.941738

**Published:** 2022-12-09

**Authors:** Jia Hu, Zhuomiao Ye, Zhe Xu, Zhinan Hao, Yongjun Wang

**Affiliations:** ^1^Department of Gastroenterology, The Second Xiangya Hospital, Central South University, Changsha, China; ^2^Research Center of Digestive Disease, The Second Xiangya Hospital, Central South University, Changsha, China; ^3^Department of Oncology, Xiangya Hospital, Central South University, Changsha, China; ^4^Department of Pharmacy, Xiangya Hospital, Central South University, Changsha, China; ^5^Department of Gastrointestinal Surgery, Hebei General Hospital, Shijiazhuang, China

**Keywords:** pembrolizumab, cost-effectiveness, *Esophageal carcinoma*, programmed death ligand-1, esophageal squamous cell carcinoma

## Abstract

**Background:**

The national Comprehensive Cancer Network has suggested pembrolizumab as a second-line therapy for esophageal squamous cell carcinoma (ESCC) patients with a programmed death ligand-1 (PD-L1) combined positive score (CPS) ≥ 10. However, despite the increased survival rate associated with pembrolizumab in these patient population, the high cost of pembrolizumab may influence its antitumor effect. This study aimed to evaluate the cost-effectiveness of pembrolizumab compared to chemotherapy as second-line treatments for esophageal carcinoma (EC) based on KEYNOTE-181 trial.

**Methods:**

A Markov model was constructed using TreeAge 2021 based on three different groups: all intent-to-treat patients (ITT population), patients with ESCC (ESCC population), and patients with a PD-L1 CPS ≥10 (CPS ≥10 population). Incremental cost, Incremental effect, Life-years, quality-adjusted life-years (QALYs) and incremental cost–effectiveness ratio (ICER) were calculated. Analyses were conducted on the setting of a willingness-to-pay threshold of $150,000 from the US perspective.

**Results:**

The ICERs for pembrolizumab were $157,589.545 per QALY, $60,238.823 per QALY, and $100,114.929 per QALY compared with chemotherapy in the ITT, ESCC, and CPS≥10 populations, respectively. The ICER of the ITT population was higher than $150,000, suggesting that pembrolizumab was not a cost-effective treatment scheme in patients with a PD-L1 CPS ≤ 10 or esophageal adenocarcinoma. The ICER was < $150,000 in the ESCC and CPS≥10 populations, indicating that pembrolizumab was cost-effective in these two subgroups.

**Conclusion:**

The determining of pembrolizumab as a cost-effective second-line therapy for EC in the United States depends on the histologic type and PD-L1 expression.

## Introduction

Esophageal carcinoma (EC) accounts for only 3.1% of all cancers but was the seventh most commonly diagnosed cancer (604,000 new cases) and the sixth highest cause of cancer-related mortality (544,000 deaths) in 2020 worldwide, which means that 1 in 18 cancer deaths in 2020 was from EC (1). EC has a poor prognosis with 5-year survival rate of only 20% in the United States (2). If diagnosed early, esophagectomy with neoadjuvant therapy is recommended for patients with locally advanced EC. However, most EC patients are diagnosed at the advanced stage because there are no specific symptoms in the early stage. Therefore, such patients have poor prognoses. Choosing a more cost-effective second-line treatment strategy should be considered when EC progresses after one treatment. Second-line treatments include chemotherapy and targeted therapies. Monoclonal antibodies that bind to the programmed death 1 (PD-1) receptor are one such targeted therapy (3).

Pembrolizumab is a monoclonal IgG4 antibody against the PD-1 receptor that has been tested in many types of malignancies, such as non-small cell lung cancer (4), and hepatocellular carcinoma (5). PD-1 is mainly expressed in T cells, including Tissue-resident memory T cells, which are resident in the gastrointestinal tract (6). So, checkpoint inhibitor is an effective therapy for gastrointestinal cancer. KEYNOTE-180 (7), a phase II clinical trial, showed that pembrolizumab can improve the prognosis of in patients with advanced EC. This trial showed that EC patients with a programmed death ligand-1 (PD-L1) combined positive score (CPS) ≥ 10 had a 13.8% objective response rate to pembrolizumab, whereas patients with a CPS < 10 only had an objective response rate of 6.3%. In addition, the objective response rate of esophageal squamous cell carcinoma (ESCC) patients treated with pembrolizumab was 14.3%, whereas that of esophageal adenocarcinoma (EAC) patients was 5.3%. These outcomes suggested that the therapeutic effect of pembrolizumab was associated with the histological type and PD-L1 expression.

KEYNOTE-181 (8), a recent phase III clinical trial, demonstrated that among patients with advanced EC, treatment with second-line pembrolizumab resulted in improved outcomes in terms of overall survival (OS) in the PD-L1 CPS≥10 population (hazard ratio, 0.69; 95% confidence interval, 0.52 to 0.83; *p* = 0.0074) and ESCC population (hazard ratio, 0.78; 95% confidence interval, 0.63 to 0.96; *p* = 0.0095) compared to chemotherapy.

In recent years, increasing attention has been paid to the cost of healthcare worldwide, and some patients are not treated with more effective strategies because of the high cost (9). Despite the increased survival rate associated with pembrolizumab in the ESCC and PD-L1 CPS ≥10 patient populations, the high cost of pembrolizumab may influence its antitumor effect. Although there was no significant increase in OS (hazard ratio,0.85;95% confidence interval, 0.72 to 1.01) in the intent-to-treat (ITT) population, because the high cost of pembrolizumab and utility values should be taken into consideration when evaluating the effect of a drug, cost-effectiveness analysis of pembrolizumab in the ITT population is also necessary. The aim of this study was to compare the cost-effectiveness of pembrolizumab with that of chemotherapy in patients with advanced EC with different subtypes.

## Methods

### Population and clinical data collection

The study was based on the KEYNOTE-181 clinical trial (8). The targeted population was patients with histologically confirmed ESCC or EAC who experienced progression after first-line therapy. The cost-effectiveness analysis was conducted in three subgroups: patients with PD-L1 CPS ≥ 10 (CPS ≥ 10 population, number = 222), ESCC patients (ESCC population, number = 401), and all ITT patients (ITT population, number = 628).

Reconstructed patient data were extracted from Kaplan-Meier curves of OS and progression-free survival (PFS), as reported in the KEYNOTE-181 trial, by Getdata Graph Digitizer (version 2.26; http://www.getdata-graph-digitizer.com). R software (version 4.0.5; http://www.r-project.org) was used to reconduct curves and fit distributions. Several common parametric models were selected to calculate the parameters as previously described (10), including the log-normal, log-logistic, gamma, Weibull, exponential, and Gompertz distributions. Based on the Akaike information criterion and Bayesian information criterion, a best-fitting parametric distribution was selected for each curve. The estimated parameters of the selected distributions for the curves are listed in Table 1.

**Table 1:** Parameters of selected models.

**Strategy**	**Treatment regimens**	**Endpoint**	**Distribution**	**Distribution information**	**AIC**	**BIC**
ITT	Chemotherapy	OS	Gamma distribution	shape = 1.4504, rate = 0.1728	1861.591	1872.84
		PFS	Log-normal distribution	meanlog = 1.27, sdlog = 0.9	1482.221	1493.47
	Pembrolizumab	OS	Log-normal distribution	meanlog = 1.95, sdlog = 1.04	1858.589	1869.837
		PFS	log-logistic distribution	shape = 1.882, scale = 2.742	1422.522	1433.77
ESCC	Chemotherapy	OS	log-logistic distribution	shape = 1.786, scale = 6.6988	1223.736	1233.691
		PFS	log-logistic distribution	shape = 1.947, scale = 3.267	956.8044	966.744
	Pembrolizumab	OS	Log-normal distribution	meanlog = 2.11, sdlog = 1.06	1193.12	1203
		PFS	log-logistic distribution	shape = 1.877, scale = 3.172	948.8619	958.6859
CPS ≥ 10	Chemotherapy	OS	Gamma distribution	shape = 1.48, rate = 0.176	684.6457	692.8805
		PFS	log-logistic distribution	shape = 1.898, scale = 3.153	535.3484	543.5832
	Pembrolizumab	OS	Log-normal distribution	meanlog = 2.20, sdlog = 0.986	648.6969	656.7154
		PFS	log-logistic distribution	shape = 1.611, scale = 3.957	530.7909	538.8267

ITT, the Intent-to-treat Population; ESCC, Patients with Squamous Cell Carcinoma; CPS ≥ 10, PD-L1, Patients with programmed cell death-Ligand 1; CPS, combined positive score ≥10; PFS, free-survival progression; OS, overall survival.

### Model structure

**Figure 1:**
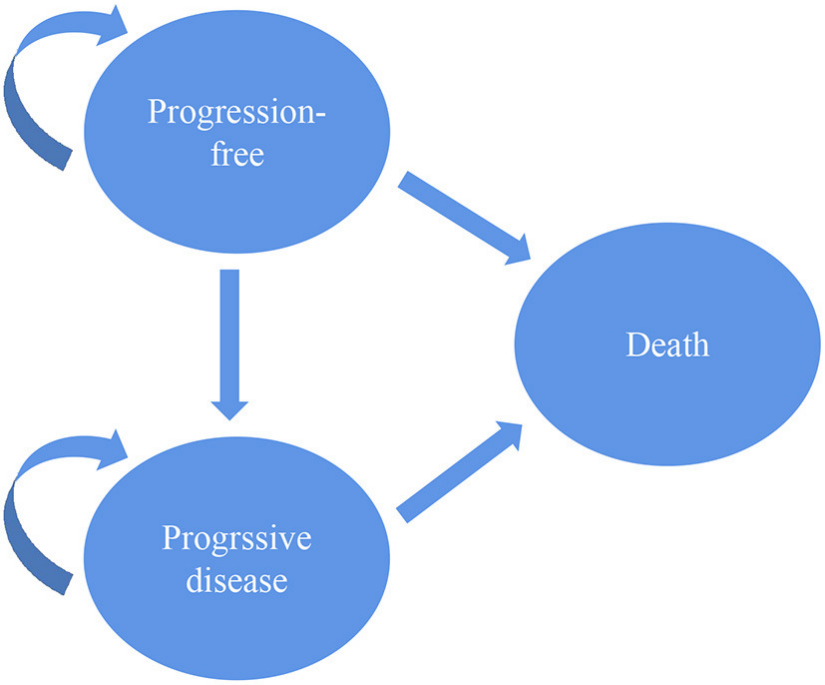
Three-state Markov model.

To evaluate the cost-effectiveness of second-line pembrolizumab for EC, a three-state Markov model was designed using TreeAge software (version 2021, Williams-town, MA; https://www.treeage.com/product/treeage-pro-healthcare). The three health states included PFS, progressive disease (PD) and death (Figure 1). All targeted populations were supposed to stay in the PFS state when they were treated with second-line pembrolizumab or chemotherapy in the first cycle. They could stay in their original state or move to other states based on transition probabilities in the following cycles. The Markov cycle was set at 3 weeks. The time horizon was set to 10 years, which was used to simulate the natural course of esophageal cancer and sufficient to model the OS of patients with advanced ESCC.

Incremental cost, Incremental effect, Life-years, quality-adjusted life-years (QALYs) and incremental cost-effectiveness ratio (ICER) were measured. Cost and effectiveness were discounted at an annual rate of 3%, as recommended by the US economic evaluation guidelines (11). The willingness-to-pay (WTP) threshold was set at $150,000 per QALY from the US third-party public healthcare payer perspective.

### Cost and utility data

Only direct medical costs, including the costs of pembrolizumab, chemotherapy, administration, follow-up, laboratory testing, best supportive care and management of grade 3–4 main adverse events (AEs) were included in the model (Table 2) (12, 14–16).

**Table 2:** Key input parameters of model.

**Variable**	**Baseline value**	**Range**		**Distribution**	**References**
		**Minimum**	**Maximum**		
**Risk for main adverse events, %**					
**Pembrolizumab group**					
Fatigue	0.6	0.48	0.72	Beta	Keynote 181 (8)
Decreased appetite	0.6	0.48	0.72	Beta	Keynote 181 (8)
Asthenia	1.3	1.04	1.56	Beta	Keynote 181 (8)
Diarrhea	0.6	0.48	0.72	Beta	Keynote 181 (8)
Anemia	1.3	1.04	1.56	Beta	Keynote 181 (8)
**Chemotherapy group**					
Diarrhea	3	2.4	3.6	Beta	Keynote 181 (8)
Anemia	7.8	6.24	9.36	Beta	Keynote 181 (8)
Neutrophil count decreased	9.8	7.84	11.76	Beta	Keynote 181 (8)
WBC count decreased	10.1	8.08	12.12	Beta	Keynote 181 (8)
Neutropenia	7.1	5.68	8.52	Beta	Keynote 181 (8)
**Health utility scores**					
Utility of PFS	0.741	0.5928	0.8892	Beta	Zhang et al. (12)
Utility of PD	0.581	0.4648	0.6972	Beta	Zhang et al. (12)
**Drug cost, $/per cycle**					
Pembrolizumab	10323.2	8258.56	12387.84	Gamma	Centers for Medicare and Medicaid Services (13)
Paclitaxel	79.5	63.6	95.4	Gamma	Centers for Medicare and Medicaid Services (13)
Docetaxel	87	69.6	104.4	Gamma	Centers for Medicare and Medicaid Services (13)
Irinotecan	61	48.8	73.2	Gamma	Centers for Medicare and Medicaid Services (13)
**Expenditures on main adverse events, $/per cycle**					
Fatigue	110.3	88.24	132.36	Gamma	Zhang et al. (12)
Decreased appetite	115.4	92.32	138.48	Gamma	Zhang et al. (12)
Anemia	508.2	406.56	609.84	Gamma	Zhang et al. (12)
Neutrophil count decreased	466	372.8	559.2	Gamma	Zhang et al. (12)
WBC count decreased	466	372.8	559.2	Gamma	Zhang et al. (12)
Neutropenia	466	372.8	559.2	Gamma	Zhang et al. (12)
Asthenia	115.4	92.32	138.48	Gamma	Chongqing et al. (14)
Diarrhea	44.6	35.68	53.52	Gamma	Li et al. (15)
**Other expenditures, $/per cycle**					
Laboratory	87.6	70.08	105.12	Gamma	Zhang et al. (12)
Follow-up per	51.5	41.2	61.8	Gamma	Zhang et al. (12)
Administration	69.81	55.85	83.77	Gamma	Ding et al. (16)
Best supportive care	117.1	93.68	140.52	Gamma	Zhang et al. (12)

In the pembrolizumab group, patients were treated with 200 mg pembrolizumab for a 3-week cycle. In the chemotherapy group, patients were treated with monotherapy according to the clinician's discretion. Clinicians selected drug among paclitaxel (80–100 mg/m^2^, three times, 4-week cycle), docetaxel (75 mg/m^2^, one time, 3-week cycle), or irinotecan (180 mg/m^2^, one time, 2-week cycle). The cost of the chemotherapy drug was assumed to be the average price of paclitaxel, docetaxel and irinotecan. When the disease progressed, the third-line agents were assumed to be single-agent paclitaxel, docetaxel, or irinotecan (17). The body surface area was assumed to be 1.86 m^2^ (18). Prices were derived from the Centers for Medicare and Medicaid Services (Table 2). To evaluate the effectiveness, we used QALYs in the model, which was adjusted by utility. Since the KEYNOTE-181 trial did not consider quality of life, utility scores of the PFS and PD states were acquired from a previously published EC-related cost-effectiveness study. Utilities of PFS and PD were set as 0.741 and 0.581, respectively (Table 2) (12).

### Sensitivity analysis

One-way sensitivity analysis and probabilistic sensitivity analysis were conducted to quantify uncertainty and assess the robustness of the Markov model. In the one-way sensitivity analysis, the parameters were set over a range of ± 20% of the baseline values (19). The results of the one-way sensitivity analysis were displayed on the tornado diagrams. In addition, probabilistic sensitivity analysis was also conducted using a Monte Carlo simulation with 10,000 iterations (20). Beta distribution was assigned for risk for main AEs and health utility values, whereas gamma distribution was assigned for cost (Table 2). Cost-effectiveness acceptability curves showed the results of the probabilistic sensitivity analysis. These curves presented the estimated probabilities of pembrolizumab and chemotherapy being regarded as cost-effective strategies at varying WTP thresholds.

## Results

### Cost-effectiveness analysis

As shown in Table 3, in the ITT population, pembrolizumab gained 0.801 QALYs at a cost of $45,829.960. The effectiveness of chemotherapy was 0.550 QALYs, and the cost was $6,332.282. Compared with chemotherapy, the mean incremental effectiveness and cost of pembrolizumab were 0.251 QALYs and $39,497.678, respectively. The ICER for pembrolizumab versus chemotherapy was $157,589.545 per QALY, although pembrolizumab when utility scores of health states were not considered, with an ICER of $91,083.929 per life-year.

**Table 3:** Cost-effectiveness analysis results.

**Strategy**	**Overall cost ($)**	**Overall LYs**	**Overall QALYs**	**Increased Cost ($)**	**Increased LYs**	**Increased QALYs**	**ICER Per LY**	**ICER Per QALY**
**ITT**								
Chemotherapy	6,332.282	0.829	0.550					
Pembrolizumab	45,829.960	1.263	0.801	39,497.678	0.434	0.251	91,083.929	157,589.545
**ESCC**								
Chemotherapy	2,575.608	0.302	0.210					
Pembrolizumab	45,261.471	1.528	0.919	42,685.863	1.227	0.709	34,800.022	60,238.823
**CPS** **≥10**								
Chemotherapy	6,309.908	0.883	0.548					
Pembrolizumab	39,022.521	1.462	0.874	32,712.613	0.579	0.327	56,480.360	100,114.929

ITT, the Intent-to-treat Population; ESCC, Patients with Squamous Cell Carcinoma; CPS ≥ 10, PD-L1, Patients with programmed cell death-Ligand 1; CPS, combined positive score ≥10.

In the ESCC population, the estimated cost of pembrolizumab was $45,261.471, which was higher than the $2,575.608 for chemotherapy, resulting in an incremental cost of $42,685.863. The pembrolizumab group gained an incremental effect of 0.709 QALYs compared with the chemotherapy group in this population. The ICER in the ESCC population was $60,238.823 per QALY, which was significantly lower than the WTP threshold.

Pembrolizumab was also more costly in the CPS ≥10 population with a cost of $39,022.521 compared with $6,309.908 for chemotherapy. Treatment with pembrolizumab yielded an incremental effect of 0.327 QALYs at an incremental cost of $32,712.613 compared with chemotherapy, resulting in an ICER of $100,114.929 per QALY. At the WTP threshold of $150,000 per QALY, pembrolizumab was also a cost-effective treatment strategy compared with chemotherapy in the CPS ≥ 10 population.

### One-way sensitivity analysis

**Figure 2:**
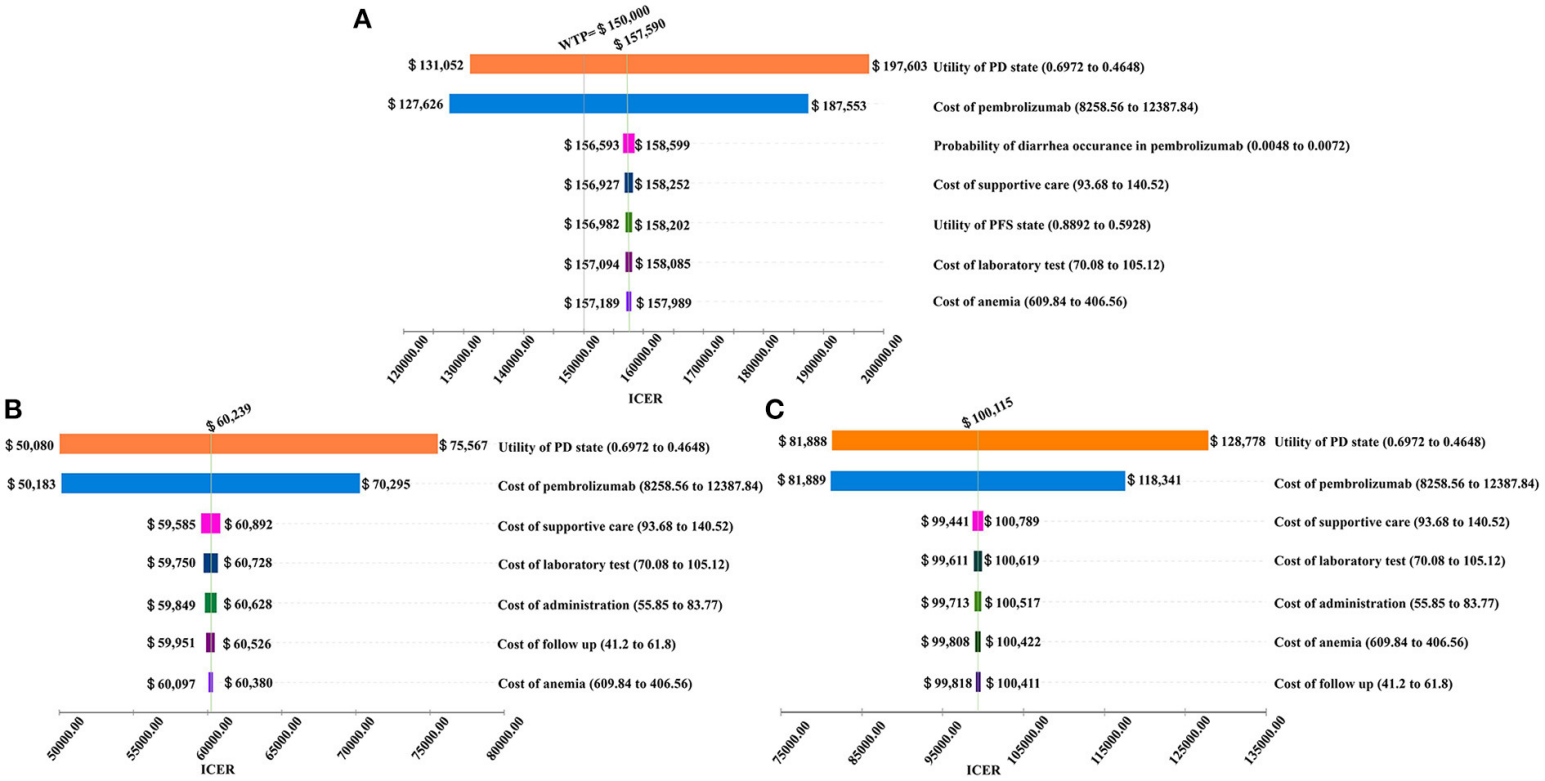
Tornado diagram for the ICER per QALY of pembrolizumab vs. chemotherapy in three groups **(A)** the Intent-to-treat Population; **(B)** Patients with Squamous Cell Carcinoma; **(C)** Patients with programmed cell death-Ligand 1 (PD-L1) combined positive score (CPS) ≥ 10.

The results of the one-way sensitivity analysis are shown in the tornado diagram (Figure 2). The utility score of the PD state was the factor that most altered the cost-effectiveness outcomes, because we focused on second-line treatment of EC, and these patients were generally in poor condition when they entered the cohort and transited to the PD state quickly. Therefore, the utility score of the PD state played a more dominant role than the utility score of the PFS state.

The cost of pembrolizumab also played a crucial role in this model. If the cost of pembrolizumab increased from $8,258.56 to $12,387.84 per cycle, the ICERs in the ITT, ESCC, CPS ≥ 10 populations increased from $127,626 to $187,553 per QALY, from $50,183 to $70,295 per QALY, and from $81,889 to $118,341 per QALY, respectively.

In addition, the probability of diarrhea occurrence in patients treated with pembrolizumab, costs of best supportive care, utility score of the PFS state, cost of laboratory tests, cost of anemia management, cost of follow-up, and cost of administration were important influential factors on the ICER.

### Probabilistic sensitivity analysis

**Figure 3:**
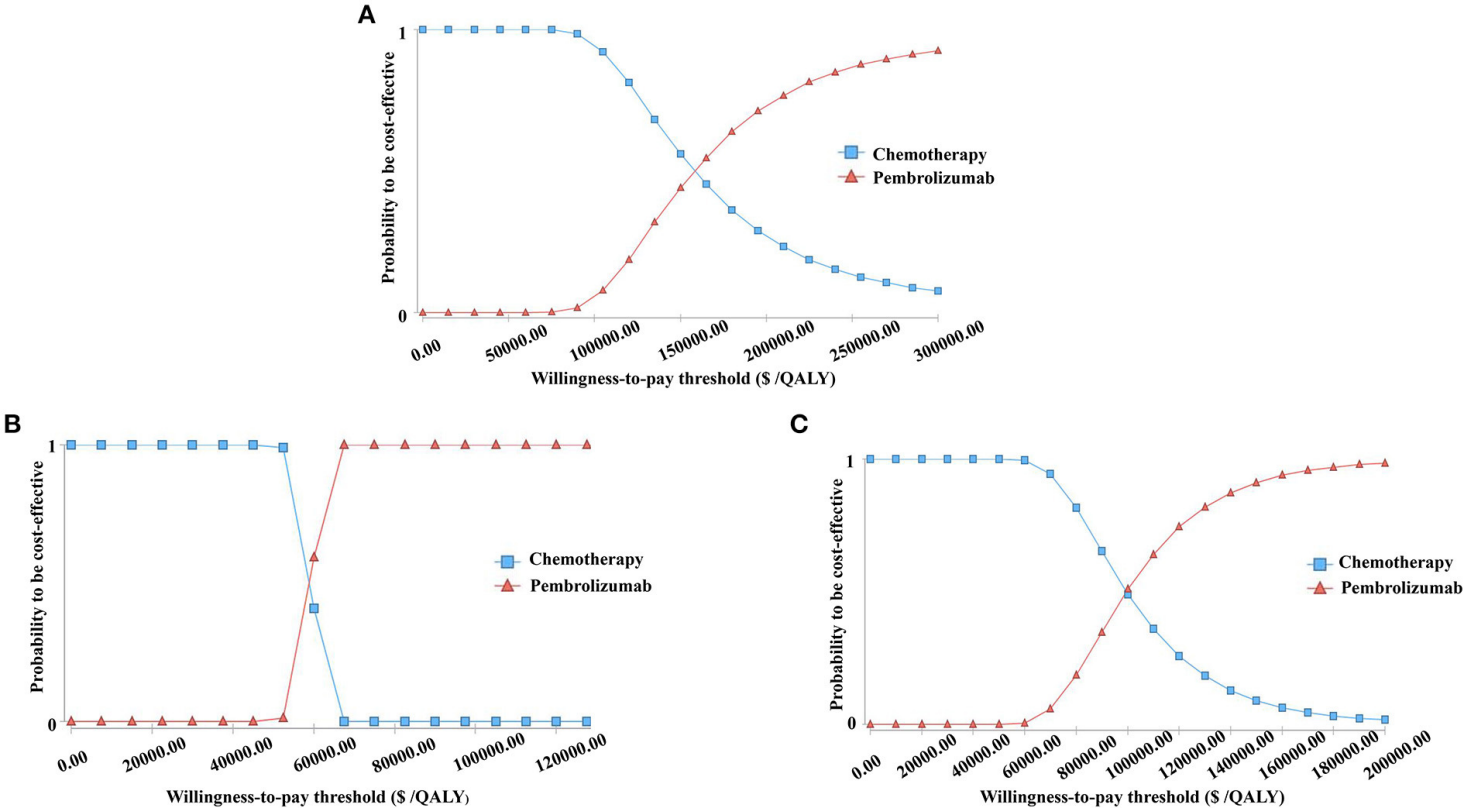
The cost-effectiveness acceptability curves for the pembrolizumab strategy compared to the chemotherapy strategy in three groups **(A)** the Intent-to-treat Population; **(B)** Patients with Squamous Cell Carcinoma; **(C)** Patients with programmed cell death-Ligand 1 (PD-L1) combined positive score (CPS) ≥ 10.

The cost-effectiveness acceptability curves are shown in Figure 3. The curves demonstrate the probability of pembrolizumab to be cost-effective compared to the WTP. At a WTP threshold of $150,000 per QALY, the cost-effectiveness acceptability curve showed that wtp, 100%, and 91.12% in the ITT, ESCC, and CPS≥10 populations, respectively. When the WTP values were about $158,000 and $99,000 per QALY in the ITT and CPS ≥ 10 populations, respectively. There was a 50% probability that pembrolizumab was a cost-effective therapy. In patients with ESCC, pembrolizumab had a 100% possibility of being a cost-effective treatment when the threshold of WTP increased to $69,000 per QALY.

## Discussion

Pembrolizumab is a representative antitumor agent that targets PD-1 and activate T cells to exert antitumor function (21). The main adverse effects of pembrolizumab are hypothyroidism and rash, which are more slight compared with chemotherapy (22). Therefore, the national Comprehensive Cancer Network has suggested pembrolizumab combined with chemotherapy as a first-line therapy for both EAC and ESCC depending on the PD-L1 expression level (23), whereas pembrolizumab only suggested in ESCC patients as a second-line therapy based on KEYNOTE-181. In my opinion, large-scale multi-center studies also should be carried out to further explore the applicable condition of pembrolizumab.

KEYNOTE-181, a phase III clinical trial, showed that pembrolizumab could prolong OS among EC patients with a PD-L1 CPS ≥ 10 and patients with ESCC compared with chemotherapy. Based on these fundings, pembrolizumab was approved by the food and drug administration of America as a second-line therapy for advanced ESCCs with CPS scores ≥ 10 (24). However, survival outcomes do not account for all aspects of a drug, and pembrolizumab contributes to a heavy burden of medical expenditure. Cost-effectiveness analysis is an indispensable part of the application of second-line pembrolizumab for EC. To our knowledge, this is the first study to examine the cost-effectiveness of pembrolizumab as a second-line treatment for EC based on subgroups of PD-L1 CPS and histopathologic type from the perspective of US.

Our results illustrated that pembrolizumab is cost-effective in the ESCC or CPS ≥10 populations, but not in the ITT population. We found that the ICER in the ITT population was $157,589.545 per QALY, which was slightly higher than the WTP of $150,000 per QALY. Pembrolizumab is also an alternative strategy for patients with high incomes and willingness to pay for health. Mei Zhan et al. reported the cost-effectiveness analysis of pembrolizumab vs. chemotherapy based on KEYNOTE-181 study from the perspective of Chinese society recently (25). They drew a diverse conclusion that compared with chemotherapy, pembrolizumab is not a cost-effective treatment option for the second-line treatment of EC in China. The ICER is $202,708.62 per QALY, $163,643.19 per QALY and $163,165.26 per QALY in the ITT population, ESCC populations and CPS ≥ 10 populations. There are several reasons for the opposite results. Firstly, we assumed that economic differences had the biggest impact on the results. Due to different economic conditions, we set WTP as the cut-off value of WTP while Mei Zhan et al. (25) set WTP according to Gross Domestic Product per capita. Secondly, though both two studies used the Markov model to evaluate the cost and QALY, the input parameters derived from relevant literature and the value could be modified basing the actual situation.

Other second-line strategies for EC have been assessed. Nivolumab is a monoclonal antibody that targets PD-1. Compared with pembrolizumab, nivolumab has the advantage of higher safety in various cancer (26). Zhang et al. (12) suggested that nivolumab is not a cost-effective option compared with chemotherapy in Chinese patients with ESCC (12). However, the cost of nivolumab is $13,674 per month based on the price derived from the Centers for Medicare and Medicaid Services (13) whereas the cost of pembrolizumab is $13,764 per month at the current price. Therefore, the price of pembrolizumab is comparable to that of nivolumab, and both prolong OS compared to chemotherapy. There are several reasons for these different results. First, given the differences in the countries' situations, different costs of drugs and WTP thresholds may be the main influential factors. Second, clinical trials demonstrated that both nivolumab and pembrolizumab could prolong OS as second-line treatment, but the median OS of chemotherapy in the two trials were different due to differences in the chemotherapy treatment strategies and demographic characteristics. Chemotherapy and immunotherapy commonly used at second-line therapy in esophageal cancer. As for ESCC patients failed first-line therapy, pembrolizumab was recommended for ESCC patients with a PD-L1 CPS ≥ 10 and nivolumab was recommended for all ESCC patients. Based on previous cost-effectiveness studies, nivolumab and pembrolizumab were found to be less cost-effective than chemotherapy on the basis of Chinese perspective. However, our study found that pembrolizumab was cost-effective in the ESCC or a PD-L1 CPS ≥10 populations from the perspective of the US. Therefore, pembrolizumab could be preferred for Americans with ESCC or a PD-L1 CPS ≥ 10 when first-line treatment of esophageal cancer failed.

In the one-way sensitivity analysis, the most sensitive parameter in the model was the utility score of the PD state, which is a measured value from experiments. The cost of pembrolizumab also largely influenced outcomes, which could be explained by its high price. Although the utility score of the PD state and cost of pembrolizumab played critical roles in the three groups, pembrolizumab was still cost-effective when changing these two values in the ESCC and CPS ≥10 populations with a WTP threshold of $150,000 per QALY. Further, when the cost of pembrolizumab was < $9,800 per 3 weeks, pembrolizumab was cost-effective even in the ITT population compared with chemotherapy. Therefore, lowering the price of pembrolizumab is a feasible strategy for further widespread second-line administration of pembrolizumab in patients for whom PD-L1 expression is not tested or patients with EAC. With the development of targeted therapy, an increasing number of PD-1 inhibitors or other molecular targeted therapeutic drugs with equal efficacy will emerge. At that time, the price of pembrolizumab may be reduced, which might make it a more cost-effective second-line treatment option in patients with EC.

Our study has some limitations. First, KEYNOTE-181 was the first randomized controlled trial of second-line pembrolizumab for EC. We used a Markov model to simulate disease progression, although some results were not detailed. For instance, KEYNOTE-181 only recorded the risk of AEs in the ITT population and did not collect these data in the ESCC and CPS ≥10 populations. Therefore, these results failed to reflect the actual situation. Second, the utility score of the PD state is a critical parameter affecting the results. However, few studies have discussed the utility scores of the PFS and PD states of EC. Existing research has always presented different utility scores. Therefore, more research should be conducted to explore the most suitable utility scores for different cancer types and health conditions. Third, utility scores of AEs were not considered in our analysis, because utility scores varied in different studies, and these utility scores lacked sufficient experimental evidence. We look forward to the publication of credible utility scores for AEs. Fourth, costs usually vary geographically and temporally due to varying economic levels, which may affect the results. However, we performed Monte Carlo simulations with 10,000 iterations to meet various requirements. Fifth, cut-off value of CPS of the PD-L1 expression was set as 10 without setting different threshold values of CPS to compare survival benefits in the KEYNOTE-181 clinical trial. A previous study indicated that advanced gastric or gastro-esophageal junction cancer patients with CPS of PD-L1 ≥ 1 treated with pembrolizumab had prolonged overall survival than total population. Results showed that cut-off value of CPS of PD-L1 was set as 1 could had a positive outcome in advanced gastric or gastro-esophageal junction cancer patients in terms of survival benefits. Further clinical studies are needed to explore the most appropriate threshold value for CPS of the PD-L1 expression. Moreover, only histological type and PD-L1 expression level were analyzed to explore the targeted population of pembrolizumab. More factors should be considered and analyzed when determine a new drug's application, such as types of precancerous lesions and human epidermal growth factor receptor 2 expression. Therefore, further cost-effectiveness analysis of esophageal carcinoma treated with pembrolizumab based on different CPS of PD-L1 expression cut-off values or other factors related to the application of pembrolizumab also should be carried out.

## Conclusion

In conclusion, pembrolizumab is cost-effective in patients with ESCC or a PD-L1 CPS ≥ 10. Therefore, pembrolizumab is another choice for the second-line treatment for patients with ESCC or a PD-L1 CPS ≥ 10 from the perspective of US health economics, in addition to current chemotherapy.

## Data availability statement

The raw data supporting the conclusions of this article will be made available by the authors, without undue reservation.

## Author contributions

JH, ZY, and YW contributed to the study design. JH and ZH contributed to data collection. JH and ZX are responsible for data curation. JH, ZY, and ZX contributed to the data analysis. JH, ZY, ZX, and ZH writing original draft. YW contributed to the revision of the manuscript. All authors approved the final manuscript.

## References

[1] Sung H Ferlay J Siegel RL Laversanne M Soerjomataram I Jemal A. Global cancer statistics 2020: globocan estimates of incidence and mortality worldwide for 36 cancers in 185 countries. CA Cancer J Clin. (2021) 71:209–49. 10.3322/caac.21660 33538338

[2] Siegel RL Miller KD Jemal A. Cancer Statistics, 2020. CA Cancer J Clin. (2020) 70:7–30. 10.3322/caac.21590 31912902

[3] Allaire JC Balk M Azmi S Handl HL Yang K Barnes G. Use of Pd-1 and Pd-L1 inhibitors after first-line therapy in esophageal cancer patients in the us. Curr Med Res Opin. (2021) 37:1403–7. 10.1080/03007995.2021.1929134 33989092

[4] Paz-Ares L Luft A Vicente D Tafreshi A Gümüş M Mazières J. Pembrolizumab plus chemotherapy for squamous non-small-cell lung cancer. N Engl J Med. (2018) 379:2040–51. 10.1056/NEJMoa1810865 30280635

[5] Finn RS Ryoo BY Merle P Kudo M Bouattour M Lim HY. Pembrolizumab as second-line therapy in patients with advanced hepatocellular carcinoma in keynote-240: a randomized, double-blind, phase III trial. J Clin Oncol. (2020) 38:193–202. 10.1200/JCO.19.01307 31790344

[6] Li P Zhang Y Xu Y Cao H Li L Xiao H. Characteristics of Cd8+ and Cd4+ tissue-resident memory lymphocytes in the gastrointestinal tract. Adv Gut Microb Res. (2022) 2022:1–12. 10.1155/2022/9157455

[7] Shah MA Kojima T Hochhauser D Enzinger P Raimbourg J Hollebecque A. Efficacy and safety of pembrolizumab for heavily pretreated patients with advanced, metastatic adenocarcinoma or squamous cell carcinoma of the esophagus: the phase 2 keynote-180 study. JAMA Oncol. (2019) 5:546–50. 10.1001/jamaoncol.2018.5441 30570649 PMC6459121

[8] Kojima T Shah MA Muro K Francois E Adenis A Hsu CH. Randomized phase III keynote-181 study of pembrolizumab versus chemotherapy in advanced esophageal cancer. J Clin Oncol. (2020) 38:4138–48. 10.1200/JCO.20.01888 33026938

[9] Dieleman JL Cao J Chapin A Chen C Li Z Liu A. Us health care spending by payer and health condition, 1996-2016. Jama. (2020) 323:863–84. 10.1001/jama.2020.0734 32125402 PMC7054840

[10] Ishak KJ Kreif N Benedict A Muszbek N. Overview of parametric survival analysis for health-economic applications. Pharmacoeconomics. (2013) 31:663–75. 10.1007/s40273-013-0064-3 23673905

[11] Sanders GD Neumann PJ Basu A Brock DW Feeny D Krahn M. Recommendations for conduct, methodological practices, and reporting of cost-effectiveness analyses: second panel on cost-effectiveness in health and medicine. Jama. (2016) 316:1093–103. 10.1001/jama.2016.12195 27623463

[12] Zhang PF Xie D Li Q. Cost-effectiveness analysis of nivolumab in the second-line treatment for advanced esophageal squamous cell carcinoma. Future Oncol. (2020) 16:1189–98. 10.2217/fon-2019-0821 32407173

[13] Centers for Medicare and Medicaid Services. Available online at: https://www.cms.gov.

[14] Chongqing T Liubao P Xiaohui Z Jianhe L Xiaomin W Gannong C. Cost-utility analysis of the newly recommended adjuvant chemotherapy for resectable gastric cancer patients in the 2011 chinese national comprehensive cancer network (NCCN) clinical practice guidelines in oncology: gastric cancer. Pharmacoeconomics. (2014) 32:235–43. 10.1007/s40273-013-0065-2 23709451

[15] Li S Peng L Tan C Zeng X Wan X Luo X. Cost-Effectiveness of Ramucirumab plus paclitaxel as a second-line therapy for advanced gastric or gastro-oesophageal cancer in China. PLoS ONE. (2020) 15:e0232240. 10.1371/journal.pone.0232240 32379763 PMC7205241

[16] Ding D Hu H Liao M Shi Y She L Yao L. Cost-effectiveness analysis of atezolizumab plus chemotherapy in the first-line treatment of metastatic non-squamous non-small cell lung cancer. Adv Ther. (2020) 37:2116–26. 10.1007/s12325-020-01292-3 32193809

[17] Ajani JA D'Amico TA Bentrem DJ Chao J Corvera C Das P. Esophageal and esophagogastric junction cancers, version 2.2019, nccn clinical practice guidelines in oncology. J Natl Compr Canc Netw. (2019) 17:855–83. 10.6004/jnccn.2019.0100 31319389

[18] Smith LS. Take a deeper look into body surface area. Nursing. (2019) 49:51–4. 10.1097/01.NURSE.0000558092.00476.db 31436723

[19] Peng Z Hou X Huang Y Xie T Hua X. Cost-effectiveness analysis of fruquintinib for metastatic colorectal cancer third-line treatment in China. BMC Cancer. (2020) 20:990. 10.1186/s12885-020-07486-w 33050905 PMC7556971

[20] Li H Lai L Wu B. Cost effectiveness of ceritinib and alectinib versus crizotinib in first-line anaplastic lymphoma kinase-positive advanced non-small-cell lung cancer. Clin Drug Investig. (2020) 40:183–9. 10.1007/s40261-019-00880-8 31820329

[21] Shen X Huang S Xiao H Zeng S Liu J Ran Z. Efficacy and safety of Pd-1/Pd-L1 Plus Ctla-4 antibodies ± other therapies in lung cancer: a systematic review and meta-analysis. Eur J Hosp Pharm. (2021). 10.1101/2020.01.08.20016915. [Epub ahead of print]. 34497128 PMC9811552

[22] Hradska K Hajek R Jelinek T. Toxicity of immune-checkpoint inhibitors in hematological malignancies. Front Pharmacol. (2021) 12:733890. 10.3389/fphar.2021.733890 34483944 PMC8414817

[23] National Comprehensive Cancer Network. Available online at: https://www.nccn.org/guidelines.

[24] The Food and Drug Administration. Available online at: https://www.nccn.org/guidelines/guidelines-detail?category=1&id=1433.

[25] Zhan M Xu T Zheng H He Z. Cost-effectiveness analysis of pembrolizumab in patients with advanced esophageal cancer based on the keynote-181 study. Front Public Health. (2022) 10:790225. 10.3389/fpubh.2022.790225 35309225 PMC8924414

[26] Xu C Chen YP Du XJ Liu JQ Huang CL Chen L. Comparative safety of immune checkpoint inhibitors in cancer: systematic review and network meta-analysis. BMJ. (2018) 363:k4226. 10.1136/bmj.k4226 30409774 PMC6222274

